# Effect of exogenous melatonin and different photoperiods on oxidative status and antioxidant enzyme activity in Chhotanagpuri ewe

**DOI:** 10.14202/vetworld.2018.130-134

**Published:** 2018-02-07

**Authors:** Pankaj Kumar Choudhary, Ajay Kumar Ishwar, Rajesh Kumar, Debasish Niyogi, Mukesh Kumar

**Affiliations:** 1 Department of Veterinary Physiology and Biochemistry, College of Veterinary Science and A.H., N.D.U.A.&T., Kumarganj, Faizabad - 224 229, Uttar Pradesh, India; 2 Department of Veterinary Physiology, Ranchi College of Veterinary Science and A.H., Birsa Agricultural University, Ranchi - 834 006, Jharkhand, India; 3 Department of Agronomy (A.H.), Bihar Agricultural University, Sabour, Bhagalpur, Bihar, India; 4 Department of Veterinary Pathology, College of Veterinary Science and A.H., N.D.U.A.&T., Kumarganj, Faizabad - 224 229, Uttar Pradesh, India; 5 Department of Veterinary Anatomy and Histology, College of Veterinary Science and A.H., N.D.U.A.&T., Kumarganj, Faizabad - 224 229, Uttar Pradesh, India

**Keywords:** catalase, Chhotanagpuri ewe, exogenous melatonin, malondialdehyde, photoperiod, superoxide dismutase

## Abstract

**Aim:**

The present study was conducted to evaluate the effect of exogenous melatonin under different photoperiods on oxidative status in Chhotanagpuri ewe.

**Materials and Methods:**

A total of 42 non-pregnant, non-lactating Chhotanagpuri ewe, having body weight ranging between 14.11±0.09 and 15.38±0.06 kg, were selected and were isolated from rams 2 months before melatonin administration. The selected animals were allocated randomly into seven groups, namely, Group I (normal control), Group II (long day [LD] control), Group III (LD+melatonin administration orally, 3 mg/day), Group IV (LD+melatonin administration subcutaneously, 1 mg/day), Group V (short day [SD] control), Group VI (SD+melatonin administration orally, 3 mg/day), and Group VII (SD+melatonin administration subcutaneously, 1 mg/day) comprising six animals in each group. Rams were then introduced into each group after completion of exogenous administration of melatonin. Blood samples with anticoagulant in vials were collected from each animal day before the start of the experiment and thereafter every month up to 5^th^ month. Hemolysate was prepared for estimation of oxidative stress parameters such as malondialdehyde (MDA), superoxide dismutase (SOD), and catalase (CAT).

**Results:**

It was observed that the level of MDA was significantly (p<0.05) higher in LD groups (Group II, III and IV) in comparison to control and SD groups (VI and VII) at 1^st^ month. MDA concentration after exogenous administration of melatonin was significantly (p<0.05) decreased in Group IV and VI in comparison to 1^st^ month. SOD was significantly (p<0.05) higher in SD groups (V, VI, and VII) at the 1^st^ month in comparison to 0 day. After exogenous administration of melatonin, SOD concentration was significantly (p<0.05) higher in Groups III and IV in comparison to 1^st^ month. CAT was significantly (p<0.05) higher in SD groups (V, VI, and VII) in comparison to control and LD groups. After exogenous administration of melatonin, CAT concentration was significantly (p<0.05) higher in Groups III, IV, VI, and VIII in comparison to Groups I, II, and V. At the 3^rd^ month, CAT concentration significantly (p<0.05) decreased in Groups III, IV, VI, and VII in comparison to 2^nd^ month of experiment. However, a decreasing trend of CAT was observed in all the groups from 3^rd^ to 5^th^ month.

**Conclusion:**

The present experiment revealed that exogenous melatonin was able to reduce significantly the level of MDA and increased the activity of SOD and CAT in Chhotanagpuri ewe.

## Introduction

In the last decade, the evaluation of oxidative stress has become increasingly important in ruminant health and animal production as complementary tools in the evaluation of the nutritional and metabolic status of the animals [1]. Sheep husbandry is the backbone of the rural economy in India. The Chhotanagpuri sheep is the only recognized breed of sheep found in Jharkhand with a few numbers in Bihar and West Bengal. This breed is maintained solely for mutton production. They produce coarse wool of poor quality. This is being utilized for carpet manufacturing. Sheep which has a vital economy in India are mostly raised under harsh environment condition as it is seasonal breeders [2]. Melatonin (N-acetyl-5-methoxy-tryptamine) was first identified and isolated from the bovine pineal extract in 1958 as a neurohormone which is synthesized and secreted mainly by the pineal gland [3]. Since its discovery, further investigation revealed that it is also produced by several other organs such as the gastrointestinal tract, brain, eye, lungs, skin, kidney, liver, thyroid, thymus, and pancreas [4]. Melatonin is an indoleamine, which is synthesized from the essential amino acid, tryptophan [5]. Its production is dependent on ambient illumination, with the release being suppressed by light. The suprachiasmatic nucleus which is the major circadian oscillator that receives light input from the retina through the retinohypothalamic tract is the one that regulates the circadian melatonin production [6]. There is an accumulation of evidence suggesting that the pattern of melatonin secretion, which is mediated by photoperiod, directly influences reproductive function.

Free oxygen radicals are created when oxygen is utilized in metabolic processes. These radicals contain “free” valence electrons, making them highly reactive, capable of causing injury to cells [7]. The term “reactive oxygen species” (ROS) not only include free radicals but also stable non-radical molecules which are capable of causing oxidation, such as hydrogen peroxide [8]. While ROS are necessary for essential physiological processes, an overabundance can result in cellular damage, commonly referred to as “oxidative stress” [9]. Anti-oxidative agents (oxygen-scavengers) are not only present endogenously but can also be administered exogenously. They reduce free radicals by donating electrons to stabilize them [10].

Research in the area of oxidative stress requires tremendous scientific attention particularly in the veterinary field. Oxidative stress needs the establishment of suitable biomarkers along with generation of their reference values for a particular species and breed. Scientists are of the opinion that a major challenge in veterinary oxidative stress research is the development of a set of blood biomarkers that can reliably and reasonably reflect the profile of oxidative stress. Field of oxidative stress is of great concern to the scientific community because of its negative impact on production and reproduction of animals. Perception of the mechanisms and reactions associated with oxidative stress can be of immense help in designing specific antioxidant therapies. The efforts to detect oxidative stress in small ruminant are still in the infancy. Keeping in the view of the above fact, the present study was designed to see the effect of exogenous melatonin administration on oxidative status and oxidative enzyme activity in small ruminants.

## Materials and Methods

### Ethical approval

The experiment followed the guidelines of the Institutional Animal Ethics Committee vide letter no. 139/528/RVC/IAEC and experimental plan followed the ethical guidelines on the proper care as well as use of the animal.

### Animals and treatment

The present study was conducted in the 42 apparently healthy, non-pregnant, non-lactating Chhotanagpuri ewes, having almost similar age between 2 and 3 years and their average body weight ranged from 14.11±0.09 to 15.38±0.06 kg reared under uniform managemental husbandry practices maintained at the Instructional Farm of Small Ruminants, College of veterinary science and A.H., Birsa Agricultural University, Kanke, Ranchi, India, located at 23.36°N latitude and 85.33°E longitude with an altitude of 651 m above mean sea level. The present experiment was conducted from February to October 2015. Mean environmental temperature, relative humidity, and photoperiod during light treatment (February) ranged between 12.7-26.8°C and 66.9-83.2%, respectively, and average photoperiod was 8.38 hrs, and during melatonin treatment (March), the corresponding values ranged between 17.0-29.9°C and 63.1-82.6%, respectively, and average photoperiod was 8.17 h. Selected ewes were isolated from rams at least 2 months before melatonin administration. The selected animals were allocated randomly into seven groups (namely, Group I to Group VII) comprising six animals in each group. Group I (Normal control): The animals in this group were exposed to normal variation in day length. Group II (long day [LD] control): In this group, in addition to natural sunlight, artificial light was provided to animal for maintaining 16-18 h of light every day for 1 month and considered as LD control. Group III (LD+melatonin administration orally, 3 mg/day): In this group, in addition to natural sunlight, artificial light was provided for maintaining 16-18 h of light every day for 1 month and after that 3 mg melatonin was given orally for 1 month to each animal. Group IV (LD+melatonin administration subcutaneously, 1 mg/day): In this group, in addition to natural sunlight, artificial light was provided for maintaining 16-18 h of light every day for 1 month and after that 1 mg melatonin was administered subcutaneously for 1 month to each animal. Group V (short day [SD] control]: Animals in this group were provided only 8 h natural daylight and were then kept in a light-proof shed for 16 h exposure in the dark every day for 1 month. This group served as SD control. Group VI (SD+melatonin administration orally, 3 mg/day): Animals in this group were provided only 8 h natural daylight and were then kept in a light-proof shed for 16 h exposure in the dark every day for 1 month and after that 3 mg melatonin was administered orally for 1 month to each animal. Group VII (SD+melatonin administration subcutaneously, 1 mg/day): Animals in this group were provided only 8 h natural daylight and they were kept in a light-proof shed for 16 h exposure in the dark every day for 1 month and after that 1 mg melatonin was administered subcutaneously for 1 month to each animal. Blood samples in anticoagulant added vials were collected from each animal day before the start of the experiment and thereafter every month up to 5^th^ month. The collected blood samples were taken immediately to the laboratory in the icebox. Hemolysate was prepared as per Placer *et al*. [11]. Anticoagulant (ethylenediaminetetraacetic acid) added to blood samples were put into centrifuge tubes and stored on ice until centrifugation at 3000 rpm for 10 min. After centrifugation, plasma and erythrocyte sediment were separated. The resultant plasma and erythrocyte sediment were pipetted off into separate tubes. Collected erythrocyte sediment was washed with 0.9% NaCl solution 3 times. The washed erythrocyte was hemolyzed with nine-fold volume of distilled water to prepare 10% red blood cell hemolysate [12], for estimation of blood antioxidant parameters.

### Observations

Membrane peroxidative damage in erythrocytes was determined in terms of malondialdehyde (MDA) production by Rehman [13]**.** The activity of superoxide dismutase (SOD) in erythrocyte lysate was determined by Marklund and Marklund [14]**.** Catalase (CAT) was assayed in hemolysate by the spectrophotometric method by Bergmeyer [15]**.**

### Statistical analysis

The data were statistically analyzed by analysis of variance test, and significance was tested at 1% and 5% level as per the method of Snedecor and Cochran [16].

## Results and Discussion

All the ewes belonging to Groups III and VI responded well and became pregnant in which melatonin was given orally. Whereas, five and four ewes became pregnant in Group IV and Group VII, respectively, in which melatonin was administered subcutaneously. In normal and LD control (GroupsI and II), three ewes became pregnant, and in SD control (Group V), two ewes became pregnant. The average of total MDA concentration in different treatment groups at different intervals is presented in Table-1. MDA was significantly (p<0.05) higher in LD groups (Groups II, III, and IV) in comparison to control and SD groups (VI and VII) at the 1^st^ month. After exogenous administration of melatonin (2^nd^ month), MDA concentration was significantly (p<0.05) lower in Groups VI and VII in comparison to Group V. MDA concentration after exogenous administration of melatonin (2^nd^ month) significantly (p<0.05) decreased in Group IV and VI in comparison to the 1^st^ month.

**Table-1:** MDA concentration (nmol/ml) of Chhotanagpuri ewes in different groups at different periods (mean±SE).

Groups	0 Day	1^st^Month	2^nd^Month	3^rd^Month	4^th^Month	5^th^Month
Group I	1.95^Bb^±0.019	1.96^BCab^±0.016	1.97^ABab^±0.016	2.01^ab^±0.030	2.04^a^±0.040	2.02^ab^±0.034
Group II	1.99^ABd^±0.019	2.03^Abcd^±0.015	2.01^Acd^±0.017	2.05^abc^±0.010	2.08^a^±0.014	2.06^ab^±0.010
Group III	1.98^ABc^±0.022	2.02^Abc^±0.019	1.98^ABc^±0.014	2.05^ab^±0.015	2.09^a^±0.014	2.07^ab^±0.015
Group IV	2.00^ABcd^±0.013	2.04^Abc^±0.011	1.99 ^Ad^±0.02	2.05^b^±0.014	2.10^a^±0.018	2.07^ab^±0.014
Group V	2.02^Aabc^±0.013	1.99^ABc^±0.016	2.01^Abc^±0.016	2.04^abc^±0.017	2.06^a^±0.023	2.05^ab^±0.016
Group VI	1.99^ABc^±0.010	1.97^BCd^±0.08	1.93^BCe^±0.004	2.03^b^±0.006	2.08^a^±0.007	2.05^b^±0.007
Group VII	1.96^ABab^±0.026	1.94^Cab^±0.023	1.92^Cb^±0.023	2.01^ab^±0.039	2.04^a^±0.046	2.02a±0.039

Means bearing different superscript vary significantly (p<0.05) within the groups (a, b, c, d, e ) and between the groups (A, B, C). MDA=Malondialdehyde, SE=Standard error

Singh *et al*. [17] reported daily variation in antioxidant enzyme activity and its possible correlation with circulating melatonin levels during two different seasons, summer (LD) and winter (SD), in Indian goat. In LD (summer), when circulating melatonin was low, MDA was high which was similar to our finding. Kumar [18] studied the effect of melatonin on MDA or lipid peroxidase in goats and observed lower MDA value in a melatonin-treated group similar to our finding. Similar antioxidative effect of melatonin has been reported by Russel *et al*. [19], Gitto *et al*. [20], Tan *et al*. [21], and Poeggeler *et al*. [22]. Kandiel *et al*. [23] reported that MDA was substantially high during ewe gestation and significant (p<0.05) high level reported at the 2^nd^ month of pregnancy. In the present study, there was an increase in MDA level at 3^rd^ and 4^th^ month might be due to pregnancy. The sheep placenta [24] has prominent roles in progesterone production early in pregnancy. Oxidative stress increases during early pregnancy because of the high metabolic rate of the placenta and increased generation of ROS [25]. The elevation of MDA during gestation in ewes would indicate an increase in lipid peroxidation in association with the rapid placental and fetal development. Such finding supports the hypothesis that pregnancy intrinsically is a state of oxidative stress arising from high placental metabolic and steroidogenic activities [26].

The average of SOD concentration in different treatment groups recorded at the different interval is presented in Table-2. SOD was significantly (p<0.05) higher in SD groups (V, VI, and VII) at 1^st^ month in comparison to 0 day, but it was non-significant (Table-2). Singh *et al*. [17] reported daily variation in antioxidant enzyme activity and its possible correlation with circulating melatonin levels during two different seasons, summer (LD) and winter (SD), in Indian goat. SOD activity had a parallel reaction with the pattern of melatonin level and had a high value when melatonin level was high, i.e., in SD winter. This direct correlation of melatonin and SOD enzyme activity has also been reported in different animal tissues and at circulating level also [27]. Our results are in agreement with the findings of Singh *et al*. [17] and Albarran *et al*. [27].

**Table-2:** SOD concentration (U/g of protein) of Chhotanagpuri ewes in different groups at different periods (mean±SE).

Groups	0 Day	1^st^Month	2^nd^Month	3^rd^Month	4^th^Month	5^th^Month
Group I	368.50^a^±4.73	367.16^Cab^±4.31	365.16^Dab^±4.14	359.33^Cabc^±5.27	350.66^ABbc^±7.15	344.33^ABc^±9.52
Group II	372.50^a^±2.51	367.66^Ca^±2.49	370.50^CDa^±2.52	362.66^BCab^±1.97	353.66^ABbc^±3.98	346.33^ABc^±6.49
Group III	369.66^ab^±3.35	364.33^Cb^±2.83	374.50^BCa^±3.07	364.16^BCb^±3.27	349.66^Bc^±3.02	337.33^Bd^±2.94
Group IV	375.00^ab^±3.34	369.33^BCb^±3.06	380.83^ABa^±2.41	371.33^ABab^±3.01	358.00^ABc^±4.75	348.66^ABc^±5.85
Group V	373.50^ab^±3.67	377.00^ABa^±3.42	375.16^ABCab^±3.54	370.00^ABabc^±3.10	363.50^Abc^±4.31	358.50^Ac^±5.98
Group VI	377.1^6b^±2.44	379.66^Aab^±2.31	384.33^Aa^±2.23	374.33^Ab^±2.21	357.00^ABc^±2.65	345.00^ABd^±2.55
Group VII	375.50^ab^±3.08	378.33^ABa^±3.10	382.66^ABa^±2.88	374.83^Aab^±3.70	363.83^Abc^±5.85	355.16^ABc^±7.86

Means bearing different superscript vary significantly (p<0.05) within the groups (a, b, c, d) and between the groups (A, B, C, D). SOD=Superoxide dismutase, SE=Standard error

After exogenous administration of melatonin (2^nd^ month), SOD concentration was significantly (p<0.05) higher in Groups III and IV and non-significantly higher in another melatonin-treated group (Groups VI and VII) in comparison to 1^st^ month. Antolin *et al*. [28] reported melatonin-elevated SOD level, due to its direct free radical scavenging activity; melatonin may be indirectly protective, as well as due to its ability to enhance the dismutation of superoxide anion radical. Our results are in agreement with the finding of Antolin *et al*. [28]. SOD level was significantly (p˂0.05) lower in Groups III and VI at the 5^th^ month in comparison to other periods, which was might be due to pregnancy in the animals of that groups. However, decreasing trend of SOD was observed in all the remaining groups from 3^rd^ to 5^th^ month (Table-2). The decrease in SOD level was not significant. Kandiel *et al*. [23] reported that SOD was considerably (p<0.05) lower in pregnant ewes as compared with cyclic ones similar with our finding. In the present study, the levels of SOD were substantially lower in pregnant ewes, and this is probably a consequence of a lower peroxidase generation. Garrel *et al*. [29] reported that the activity of SOD2 (located in mitochondria) significantly decreased in sheep placentomes during the period of most rapid placental growth and high steroidogenic activity (from days 35 to 55). Theoretically, an elevated activity of SOD2 would promote H_2_O_2_ production and propagation of highly reactive ROS in the mitochondria and cytoplasm [29].

The initial concentration of CAT concentration in different treatment group recorded at different intervals is presented in Table-3. CAT was significantly (p<0.05) higher in SD groups (V, VI, and VII) in comparison to control and LD groups. After exogenous administration of melatonin (2^nd^ month), CAT concentration was significantly (p<0.05) higher in Groups III, IV, VI, and VIII in comparison to Groups I, II, and V. At the 3^rd^ month, CAT concentration significantly (p<0.05) decreased in Groups III, IV, VI, and VII in comparison to 2^nd^ month of experiment. However, a decreasing trend of CAT was observed in all the groups from 3^rd^ to 5^th^ month (Table-3).

**Table-3:** CAT concentration (mM/min/mg of protein) of chhotanagpuri ewes in different groups at different periods (mean±SE).

Groups	0 Day	1^st^Month	2^nd^Month	3^rd^Month	4^th^Month	5^th^Month
Group I	63.91^Ca^±1.12	63.56^Ba^±0.98	62.97^Dab^±0.97	60.43^Dabc^±1.52	58.74^Dbc^±1.97	57.21^Dc^±2.42
Group II	64.93^ABCa^±0.41	63.24^Bab^±0.25	63.93^CDab^±0.31	61.62^CDbc^±0.66	60.22^CDcd^±1.09	59.21^CDcd^±1.38
Group III	64.48^BCcd^±0.91	63.06^Bd^±0.82	71.71^Ba^±0.66	67.71^Bb^±0.70	65.89^Bbc^±0.66	63.95^ABcd^±0.73
Group IV	65.55^BCcd^±0.61	64.18^Bd^±0.58	72.52^ABa^±0.55	68.98^ABb^±0.92	67.20^ABbc^±1.14	65.33^ABcd^±1.41
Group V	65.39^BCab^±0.60	65.97^Aa^±0.53	65.21^Cab^±0.47	63.63^Cbc^±0.56	62.53^Cc^±0.89	61.68^BCc^±1.24
Group VI	66.51^Ad^±0.19	67.04^Acd^±0.21	73.31^Aa^±0.18	69.80^ABb^±0.13	67.20^ABc^±0.27	65.04^ABe^±0.27
Group VII	66.10^ABd^±0.27	67.12^Ad^±0.32	73.34^Aa^±0.20	70.97^Ab^±0.51	69.28^Abc^±0.92	67.81^Acd^±1.30

Means bearing different superscript vary significantly (p<0.05) within the groups (a, b, c, d, e) and between the groups (A, B, C, D). CAT=Catalase, SE=Standard error

The CAT acts on the H_2_O_2_ produced by the action of SOD on the free radicals and converts it into non-toxic products. CAT showed a complementary relationship with the activity of SOD [17]. Hence, SOD and CAT activity had a parallel relation with the pattern at melatonin levels and had a high value when melatonin level was high and low when melatonin was less [17]. Tan *et al*. [21] reported that melatonin scavenges H_2_O_2_ with the formation of N1-acetyl-N2-formyl-5-methoxykynuramine (AFMK). When AFMK was incubated with CAT, the enzyme catalytically converted AFMK to AMK could be free radical scavengers and suggested cascade, which could increase the efficiency of melatonin as an antioxidant.

Initially up to 2 months of the experimental period, all the used animals for the experimental purpose were non-pregnant female. Hence, there is no effect of pregnancy in altering the result on oxidative stress up to 2 months of experimental study. There may be minor variation in the result of oxidative stress due to pregnancy, but the pregnant animals were not discarded in the present study as the study was also done to assess the effect of exogenous melatonin on the reproductive performance of the ewe. Further, the results in the present study also give an indication in decreasing the oxidative stress by the exogenous melatonin even after a variable percentage of pregnancy in different groups after 2 months of experimental period.

## Conclusion

From the present study, it can be concluded that exogenous melatonin was able to reduce significantly the level of MDA and increase the activity of SOD and CAT in Chhotanagpuri ewe.

## Authors’ Contributions

PKC and AKI have conceived planned and designed the study and conducted the research. PKC, AKI, and RK analyzed and kept a due record of the data. Manuscript framed and drafted by PKC, AKI, DN, and MK. All authors read and approved the final manuscript.
